# Prevalence of diabetes and diabetic macular edema in patients undergoing senile cataract surgery in Italy: The DIabetes and CATaract study

**DOI:** 10.1177/1120672119830578

**Published:** 2019-03-11

**Authors:** Giacomo Panozzo, Giovanni Staurenghi, Giulia Dalla Mura, Diana Giannarelli, Giovanni Alessio, Salvatore Alongi, Romolo Appolloni, Antonio Baldascino, Francesco Boscia, Aldo Caporossi, Matteo Cereda, Erminia D’Ugo, Matteo Fallico, Teresio Avitabile, Alessandro Galan, Carlo La Spina, Giuseppe Lo Giudice, Leonardo Mastropasqua, Carmela Palmisano, Claudio Panico, Maria Cristina Parravano, Rachele Penna, Pierpaolo Pintore, Agostino Vaiano, Michele Reibaldi, Stanislao Rizzo, Tommaso Rossi, Monica Varano, Gianni Virgili

**Affiliations:** 1 ESASO European School of Advances Studies in Ophthalmology, Lugano, Switzerland; 2 Department of Biomedical and Clinical Science Sacco Hospital, University of Milan, Italy; 3 Department of biostatistics, Regina Elena National Cancer Institute-IRCCS, Rome, Italy; 4 Department of Ophthalmology, University of Bari, Italy; 5 Ophthalmology Unit, Rapallo General Hospital, Italy; 6 Ophthalmology Unit, Sant’Eugenio General Hospital, Rome, Italy; 7 Department of Ophthalmology, Policlinico Gemelli, Roma, Italy; 8 Department of Ophthalmology, University of Sassari, Italy; 9 Department of Ophthalmology, University of Chieti, Italy; 10 Department of Ophthalmology, University of Catania, Italy; 11 Ophthalmology Unit, Sant’Antonio General Hospital, Padova, Italy; 12 Ospedale Oftalmico, Torino, Italy; 13 Fondazione GB Bietti, Roma, Italy; 14 Ophthalmology Unit, Cuneo General Hospital, Italy; 15 Department of Ophthalmology, University of Firenze, Florence, Italy; 16 Ophthalmology Unit, Policlinico San Martino, Genova, Italy

**Keywords:** Diabetes, cataract, cataract surgery, phacoemulsification, diabetic macular edema, diabetic retinopathy

## Abstract

**Background:**

The purpose of this study is to determine the prevalence of diabetes and diabetic macular edema in patients undergoing senile cataract surgery in Italy.

**Methods:**

It is a prospective, multicenter, cross-sectional study. Thirteen ophthalmic units equally distributed across the Italian territory have been involved in the study. For a period of 3 months, all subjects undergoing phacoemulsification received an Optical Coherence Tompgraphy (OCT) scan and were screened for the anamnestic presence of diabetes. In addition, five selected units collected blood samples from all their patients to measure glycated hemoglobin (HbA1c) levels and detect the presence of occult diabetes (HbA1c > 6.5%). In diabetic patients, levels of retinopathy were measured and diabetic macular edema was considered significant (clinically significant macular edema) when foveal thickness was above 30% of normal levels.

**Results:**

A total number of 3657 subjects have been screened. Among them, 20.4% were diabetics. Prevalence of diabetes was significantly higher in males (24.7%) than in females (17%). Levels of HbA1c were tested in a representative sample of 1216 consecutive subjects, and occult diabetes was diagnosed in 4.8% of cases. No significant differences were observed between age groups or different geographic areas. Among diabetic patients, diabetic macular edema of any kind was present in 27.5% (clinically significant macular edema (6.6%)). No significant differences were seen in the prevalence of diabetic macular edema between males and females or between age groups. Among the 745 diabetic patients, no signs of retinopathy were seen in 537 subjects (76.3%), while 101 patients (14.3%) had nonproliferative retinopathy, 13 (1.7%) had nontreated proliferative diabetic retinopathy, and 53 (7.5%) had laser-treated retinopathy. In the entire sample of 3657 subjects, a normal macula was present in 90.9% of cases, diabetic macular edema of any kind in 5.4%, and other maculopathies in 3.4%.

**Conclusion:**

In this large cohort study on patients undergoing cataract surgery, more than one-fourth were diabetics and more than one-fourth of these had diabetic macular edema. These high prevalences suggest the opportunity to plan an adequate preoperative assessment in all patients in order to reduce the risk of postoperative development or worsening of a sight-threatening complication such as chronic diabetic macular edema.

## Introduction

Diabetes mellitus (DM) is presently estimated to affect about 8.5% of the overall population in Europe,^1^ and its prevalence increases with age, rising to over 20% in males and 15% in females aged 65 years or older.^2,3^ Diabetes is well known to adversely affect all ocular tissues, including the crystalline lens. Chronic hyperglycemia leads to the production of advanced glycation end products, increased oxidative stress, and increased activation of the polyol pathway, each of which has been implicated in the development of cataracts.^4–6^ As a result, cataract develops and progresses more frequently, rapidly, and at an earlier age in patients with diabetes. The risk of cataract development in DM is fivefold higher than in the general population, and cataract is diagnosed twice as frequently in diabetic subjects.^7,8^

At present, cataract surgery is the most commonly performed surgical procedure in Western countries,^9^ and diabetics represent a sizable percentage of surgical patients. Although phacoemulsification dramatically decreases the risk of intra- or postoperative complications, cataract surgery in diabetic patients still represents an inflammatory insult that may potentially be associated with worsening of retinopathy and may lead to the development or worsening of macular edema, with a progressive risk correlated with the level of retinal microvascular insult and stage of retinopathy intra or postoperative.^10–12^

Patients with diabetes should therefore be identified and adequately managed in order to decrease the risk of complications that may potentially diminish or even negate the benefits of cataract surgery.

The aim of the present study (named DIabetes and CATaract—DICAT study) was to assess the prevalence of diabetic patients and their level of retinopathy and macular edema in a routine setting of subjects undergoing cataract surgery in Italy.

## Materials and methods

Since the aim of the present study was to obtain representative data of the current scenario in Italy, only ophthalmology departments operating within the Public Healthcare System were considered. Among these centers, we identified those who adopted a preoperative protocol routinely including information on the presence of diabetes and OCT examination of the macular region. A total of 13 units, uniformly distributed throughout the country, met these criteria and, after IRB approval for data collection and transmission to the coordinating center, agreed to participate in the study.

Each of the 13 units prospectively and consecutively collected their data on all patients aged over 54 who were undergoing phacoemulsification with intraocular lens implantation for senile cataract surgery for a 3-month period during the first half of year 2018.

**Figure 1.**
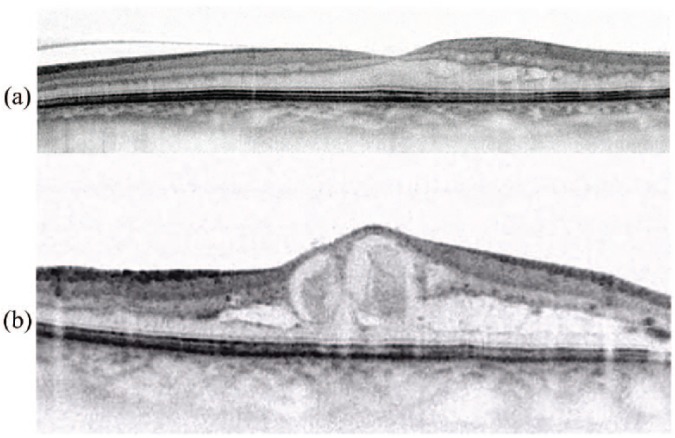
(a) Nonclinically significant macular edema (N-CSME): Presence of intraretinal cysts associated with central foveal thickness (CFT) of 257 µm (thickening <30% compared to normal values). (b) Clinically significant macular edema (CSME): Presence of intraretinal cysts associated with CFT of 598 µm (thickening >30% compared to normal values).

Data were collected using a standardized form containing the following parameters and categories:

1. Gender.
2. Age: Two groups were considered—55–70 and >70 years.
3. Presence and duration of diabetes, as reported by the patients with and stated in preoperative questionnaire filled out by the patient’s general practitioner.
4. Glycated hemoglobin (HbA1c). Five of the 13 units routinely tested it in all their patients to detect previously undiagnosed diabetes. Two groups were considered, above or below 48 mmol/mol (6.5%) as per international guidelines for the diagnosis of diabetes.
5. Presence of diabetic retinopathy (see Table 1):
  a. None
  b. Nonproliferative diabetic retinopathy (NPDR)
  c. Proliferative diabetic retinopathy (PDR)
  d. Laser-treated retinopathy.
6. Macular edema: A Spectral-Domain OCT (SD-OCT) scan of the macular area was obtained with the OCT model routinely used at each unit by radial scan (at least eight radial scans) or raster scan (at least eight horizontal scans of the macular area). Four categories were considered:
  a. Macula within normal limits.
  b. Non-clinically significant macular edema (N-CSME): Presence of intraretinal cysts associated with central foveal thickness (CFT) within normal limits or with thickening <30% compared to current standards for each OCT model (Figure 1(a)).
  c. Clinically significant macular edema (CSME): Presence of intraretinal cysts associated with foveal thickening >30% compared to current standards for each OCT model (Figure 1(b)).
  d. Presence of intra- or subretinal cysts or other lesions of dubious nature and/or associated with maculopathy of nondiabetic origin.

**Table 1.** Classification of DR based on morphologic retinal lesions.^13^

Retinal lesions	Classification
Absent	No retinopathy
Rare microaneurysms and retinal hemorrhages	Mild NPDR
Microaneurysms Retinal hemorrhages Hard exudates Cotton wool spots Not associated to lesions of advanced NPDR (see below)	Moderate NPDR
Numerous retinal hemorrhages Numerous cotton wool spots IRMA Venous beading	Advanced NPDR
New vessels at the optic disc and/or retina Preretinal hemorrhages Fibro-glial membranes	PDR

DR: diabetic retinopathy; NPDR: nonproliferative diabetic retinopathy; IRMA: intraretinal microvascular abnormality; PDR: proliferative diabetic retinopathy.

In the case of patients operated on both eyes during the data collection period, only the first eye was considered.

Each unit sent their database every 2 weeks to the coordinating center. Data were then checked for consistency and validated.

For analysis, data were finally assembled into four macro-areas representative of the Italian territory: North, Central, South, and Islands.

### Statistical analysis

Prevalence rates were calculated as the ratio of the number of eyes with diagnosis of diabetes to the number of eyes submitted to cataract surgery. Each estimated prevalence rate was associated with its 95% confidence interval to consider sampling error. The study was designed to reach a sample size of 3000 eyes in order to maintain a standard error <1%. Prevalence rates were also estimated in subgroups with sufficient sample size. Differences in prevalence rates between subgroups of eyes were evaluated using a chi-square test; *p* values <0.05 were considered statistically significant.

## Results

Demographics are reported in Table 2. A total number of 3657 subjects undergoing cataract surgery were analyzed, equally distributed across geographic areas; 71% were over 70 years of age, with a slight prevalence for females (56.5% vs 43.5%).

**Table 2.** Summary of data on subjects undergoing senile cataract surgery at the 13 centers.

Subjects	Italy (%)	North (%)	Central (%)	South (%)	Islands (%)
Total	3657	1040	946	1116	555
55–70	1057 (28.9)	276 (26.5)	261 (27.6)	324 (29.0)	196 (35.3)
>70	2600 (71.1)	764 (73.5)	685 (72.4)	792 (71.0)	359 (64.7)
Males	1590 (43.5)	397 (38.2)	410 (43.3)	510 (45.7)	273 (49.2)
55–70	466 (12.7)	110 (10.6)	122 (12.9)	137 (12.3)	97 (17.5)
>70	1124 (30.7)	287 (27.6)	288 (30.4)	373 (33.4)	176 (31.7)
Females	2067 (56.5)	643 (61.8)	536 (56.7)	606 (54.3)	282 (50.8)
55–70	591 (16.2)	166 (16.0)	139 (14.7)	187 (16.8)	99 (17.8)
>70	1476 (40.4)	477 (45.9)	397 (42.0)	419 (37.5)	183 (33.0)

The prevalence of diabetes is reported in Table 3. Among the 3657 subjects observed, 745 were diabetics, with a prevalence of 20.4%. Mean duration of diabetes in subjects below 70 years of age was 11.4 years for males and 9.2 years for females, while in subjects over 70, it was 15.9 years for males and 13.5 years for females. There was no significant difference between age groups or geographic areas, although the prevalence of diabetes was significantly higher in males (24.7%) than in females (17%), and this difference was confirmed in each age group. While the prevalence of diabetes in males did not statistically differ between the two age groups, older females presented a statistically higher prevalence (18.5% vs 13.4%, *p* < 0.0001).

**Table 3.** Summary of patients with and without diabetes by geographic area.

	Patients (% of total)	Patients with diabetes	Prevalence of diabetes (95% CI)	*p* value
All	3657	745	20.4 (19.1–21.7)	
Gender				<0.0001
Male	1590 (43.5)	393	24.7 (22.6–26.8)	
Female	2067 (56.5)	352	17.0 (15.4–18.6)	
Age				0.08
55–70	1057 (28.9)	196	18.5 (16.2–20.9)	
>70	2600 (71.1)	549	21.1 (19.5–22.7)	
Gender/age				<0.0001
Male 55–70	466 (12.7)	117	25.1 (21.2–29.0)	
Female 55–70	591 (16.2)	79	13.4 (10.6–16.1)	
Male >70	1124 (30.7)	276	24.6 (22.0–27.1)	
Female >70	1476 (40.4)	273	18.5 (16.5–20.5)	
Geographic area				0.38
North	1040 (28.4)	220	21.1 (18.7–23.6)	
Center	946 (25.9)	175	18.5 (16.0–21.0)	
South	1116 (30.5)	238	21.3 (18.9–23.7)	
Islands	555 (15.2)	112	20.2 (16.8–23.5)	

CI: confidence interval.

Levels of HbA1c were tested in a total of 1216 consecutive subjects. Among these, HbA1c was <48 mmol/mol (6.5%) in 1042 cases, and higher in 174 cases (14.3%), but only 116 of these patients were “overt ” diabetics, while in 58 subjects (4.8%), this test revealed an “occult diabetes.”

Table 4 reports OCT findings. In the whole sample of 3657 subjects, a normal macula was present in 90.9% of cases, diabetic macular edema (DME) of any kind in 5.4%, and other maculopathies in 3.4%.

**Table 4.** Summary of OCT macular findings.

OCT findings	No. of patients (%)	95% CI
Diabetic patients (*n* = 745)		
N-CSME	156 (20.9%)	18.0–23.9
CSME	49 (6.6%)	4.8–8.4
N-CSME + CSME	205 (27.5%)	24.3–30.7
No DME	540 (72.5%)	69.3–75.7
Overall population (*n* = 3657)		
Normal macula	3326 (90.9%)	90.9–91.9
DME	205 (5.4%)	4.6–6.2
Other maculopathies	126 (3.4%)	2.9–4.0

OCT: Optical Coherence Tomography; CI: confidence interval; N-CSME: nonclinically significant macular edema; CSME: clinically significant macular edema; DME: diabetic macular edema.

Among diabetic subjects, 72.5% (540 cases) had a normal macula, while DME of any kind was present in 205 cases (27.5%), of whom 156 cases of N-CSME (20.9%) and 49 cases of CSME (6.6%). No significant differences were seen in the prevalence of DME between males and females (*p* = 0.41) or between the two age groups (*p* = 0.59). However, in diabetic patients, the prevalence of DME was significantly higher in males than in females (*p* < 0.0001) and was also higher in the younger group (*p* = 0.0006).

Table 5 reports the grade of diabetic retinopathy. No signs of retinopathy were seen in 537 patients (72%), while 142 patients (19%) had NPDR, 13 (1.7%) had untreated PDR and 53 (7.1%) were already laser treated.

**Table 5.** Grade of diabetic retinopathy.

	No. of patients (%)	95% CI
Grade 1 (no retinopathy)	537 (72%)	69.8–75.3
Grade 2 (NPDR)	142 (19%)	16.7–22.1
Grade 3 (PDR)	13 (1.7%)	0.9–2.8
Grade 4 (laser-treated retinopathy)	53 (7.1%)	5.6–9.5

CI: confidence interval; NPDR: nonproliferative diabetic retinopathy; PDR: proliferative diabetic retinopathy.

## Discussion

The development of cataract is more frequent and progresses more rapidly in patients with diabetes compared to the general population. Phacoemulsification greatly reduces the risk of postoperative inflammatory complications in these patients, particularly the development of macular edema, and some authors did report no differences between diabetic eyes without preoperative DME and reference groups. These reports were, however, recently denied by a large study from the United Kingdom on 81,984 eyes undergoing cataract extraction. This study reported that in a sample of 4485 diabetic eyes, even eyes with no retinopathy had an increased risk ratio (RR) of postoperative macular edema of 1.80 compared with the reference cohort. This RR increased to a maximum of 10.34 with escalating severity of diabetic retinopathy and did not resolve even in eyes with panretinal photocoagulation.^14^ In the presence of preoperative DME, the risk of worsening becomes particularly marked, potentially diminishing or even vanishing the benefits of cataract removal on visual acuity.^15,16^ In consideration of the above, it would be crucial to identify patients with diabetes and their grade of retinopathy and maculopathy prior to cataract surgery so that they can benefit from adequate therapeutic preventive measures to reduce the risk of postoperative complications.^17^

This study emphasizes the high prevalence of diabetes in a significant and representative cohort of 3657 patients collected in a routine surgical cataract setting in Italy, and provides new and interesting insight into the prevalence of two different stages of DME (CSME and N-CSME) in these patients.

This study highlights some relevant clinical data: more than a quarter of patients undergoing cataract surgery are diabetic, and a quarter of them already show some form of DME, putting all these subjects at an increased risk of developing sight-threatening complications. This means that in Italy, where more than 500,000 cataract surgeries are performed every year, more than 120,000 procedures are most probably performed in diabetic patients yearly, and 40,000 in patients with preoperative DME.

The overall prevalence of diabetes was 25.2% (diagnosed diabetes 20.4% and undiagnosed diabetes 4.8%). Compared to the prevalence of diabetes in Italy in the same age bracket, our population reveals a higher occurrence of diabetes (25.2% vs 17.8%), probably due to the higher incidence of cataract among diabetic patients.^18^ The prevalence of undiagnosed diabetes in our study was 4.8%, which is lower than 9% reported by Feldman-Billard et al.^19^ in a smaller but still relevant study on 137 patients.

DME of any kind was present in more than a quarter of diabetic patients (27.5%), corresponding to 5.4% of the total population of this study. The vast majority of these cases were N-CSME, defined as DME with macular thickening less than 30% above normal foveal thickness, while only 6.6% were classified as CSME. Although research on this specific issue is limited, we may presume that due to the already established retinal microvascular impairment, even N-CSME is associated with a higher risk of worsening and consequent visual loss compared to eyes without preoperative DME. A further study following up these eyes after surgery is ongoing.

There are limited data in the literature with which our findings can be compared, and, to our knowledge, there are no reports on the prevalence of diabetes and DME prospectively collected in a regular clinical setting. A very large retrospective database on more than 80,000 electronic medical records from the United Kingdom reported that 21.8% of eyes undergoing cataract surgery were diabetics, and that in these eyes, the presence of “any signs of maculopathy (DME or other)” was 1.8%. These data are unreliable data because in almost 7000 cases, macular status was not recorded.^14^

Due to the generic design of the survey, as was required in order to collect consecutive and reliable data from high-volume cataract surgery centers, this study has several limitations. For instance, even following previous precise instructions and performing continuous monitoring, data were collected directly at each participating center and then sent to the coordinating center, thus possibly decreasing the homogeneity of the defined categories. Moreover, the study lacks information on type of diabetes, diabetic therapy, and glycemic control, and on separation between naïve and chronic DME with no records on previous therapy.

Current international guidelines for extraction of cataracts, although stressing the need to identify any risk factors for the development of intra- or postoperative complications, do not provide specific indications for DM and severity retinopathy and/or maculopathy, leaving this issue to the preferences of each individual center.^20^ Nevertheless, the prevalence rate of diabetes and DME found in this study strongly indicates that the issue warrants greater attention, and encourages to consider a stricter preoperative assessment, in order to adopt proper therapeutic measures to reduce the risk of developing or worsening of a sight-threatening complication such as chronic DME.

## Supplemental Material

Supplemental_Material – Supplemental material for Prevalence of diabetes and diabetic macular edema in patients undergoing senile cataract surgery in Italy: The DIabetes and CATaract study Click here for additional data file. Supplemental material, Supplemental_Material for Prevalence of diabetes and diabetic macular edema in patients undergoing senile cataract surgery in Italy: The DIabetes and CATaract study by Giacomo Panozzo, Giovanni Staurenghi, Giulia Dalla Mura, Diana Giannarelli, Giovanni Alessio, Salvatore Alongi, Romolo Appolloni, Antonio Baldascino, Francesco Boscia, Aldo Caporossi, Matteo Cereda, Erminia D’Ugo, Matteo Fallico, Teresio Avitabile, Alessandro Galan, Carlo La Spina, Giuseppe Lo Giudice, Leonardo Mastropasqua, Carmela Palmisano, Claudio Panico, Maria Cristina Parravano, Rachele Penna, Pierangelo Pintore, Agostino Vaiano, Michele Reibaldi, Stanislao Rizzo, Tommaso Rossi, Monica Varano and Gianni Virgili in European Journal of Ophthalmology
