# Supplementary material for: Prevalence of diabetes and diabetic macular edema in patients undergoing senile cataract surgery in Italy: The DIabetes and CATaract study
Source: Eur J Ophthalmol. 2019 Mar 11;30(2):315–20. doi: 10.1177/1120672119830578 (PMC7079292; doi:10.1177/1120672119830578)
Supplement: Supplemental_Material – Supplemental material for Prevalence of diabetes and diabetic macular edema in patients undergoing senile cataract surgery in Italy: The DIabetes and CATaract study [file Supplemental_Material.pdf]

Supplementary material

List of Italian Ophthalmic Centers involved in the DICAT Study

1. Ophthalmic Hospital, Torino.
2. Ophthalmology Unit, General Hospital, Cuneo.
3. Department of Biomedical and Clinical Science, University of Milan.
4. Ophthalmology Unit, Sant'Antonio General Hospital, Padova.
5. Ophthalmology Unit, Policlinico San Martino, Genova
6. Department of Ophthalmology, University of Florence.
7. Ophthalmology Unit, Sant'Eugenio General Hospital, Rome.
8. Department of Ophthalmology, Policlinico Gemelli, Rome.
9. Fondazione GB Bietti, Rome.
10. Department of Ophthalmology, University of Chieti.
11. Department of Ophthalmology, University of Bari.
12. Department of Ophthalmology, University of Catania.
13. Department of Ophthalmology, University of Sassari.
